# Emergency department experiences of people who use drugs who left or were discharged from hospital against medical advice

**DOI:** 10.1371/journal.pone.0282215

**Published:** 2023-02-23

**Authors:** Samara Mayer, Verena Langheimer, Seonaid Nolan, Jade Boyd, Will Small, Ryan McNeil

**Affiliations:** 1 British Columbia Centre on Substance Use, Vancouver, British Columbia, Canada; 2 Interdisciplinary Studies Graduate Program, University of British Columbia, Vancouver, British Columbia, Canada; 3 Department of Psychiatry, University of British Columbia, Vancouver, British Columbia, Canada; 4 Centre for Health Evaluation and Outcome Sciences, Vancouver, British Columbia, Canada; 5 Department of Medicine, University of British Columbia, Vancouver, British Columbia, Canada; 6 Faculty of Health Sciences, Simon Fraser University, Burnaby, British Columbia, Canada; 7 Centre for Applied Research in Mental Health and Addiction, Vancouver, British Columbia, Canada; 8 Internal Medicine, Yale School of Medicine, New Haven, Connecticut, United States of America; 9 Yale Program in Addiction Medicine, Yale School of Medicine, New Haven, Connecticut, United States of America; University of Toronto Leslie Dan Faculty of Pharmacy, CANADA

## Abstract

**Background:**

People who use drugs (PWUD) frequent emergency departments at a higher rate than the general population, and experience a greater frequency of soft tissue infections, pneumonia, and chronic conditions such as, HIV/AIDs and hepatitis C. This population has distinct health care considerations (e.g. withdrawal management) and are also more likely to leave or be discharged from hospital against medical advice.

**Methods:**

This study examines the experiences of PWUD who have left or been discharged from hospital against medical advice to understand the structural vulnerabilities that shape experiences with emergency departments. Semi-structured qualitative interviews were conducted with 30 PWUD who have left or been discharged from hospital against medical advice within the past two years as part of a larger study on hospital care and drug use in Vancouver, Canada.

**Results:**

Findings characterize the experiences and perceptions of PWUD in emergency department settings, and include: (1) stigmatization of PWUD and compounding experiences of discrimination; (2) perceptions of overall neglect; (3) inadequate pain and withdrawal management; and (4) leaving ED against medical advice and a lack of willingness to engage in future care.

**Conclusions:**

Structural vulnerabilities in ED can negatively impact the care received among PWUD. Findings demonstrate the need to consider how structural factors impact care for PWUD and to leverage existing infrastructure to incorporate harm reduction and a structural competency focused care. Findings also point to the need to consider how withdrawal and pain are managed in emergency department settings.

## Introduction

People who use drugs (PWUD) have complex health needs, including: HIV/AIDS [1], hepatitis C [2], mental illness [3], soft tissue infections [4, 5], and/or non-fatal overdose events [6]. Accordingly, PWUD frequent emergency department (ED) settings at high rates [7–11]. Epidemiological and cohort focused studies have investigated social-structural factors, such as homelessness [9], poverty [9, 10], and incarceration [10] that drive ED visits amongst PWUD, as well as racial disparities associated with such structural factors [9, 10]. However, there has been less attention paid to social-structural dynamics within ED settings that shape the experiences and perspectives of PWUD despite high rates of ED utilization [10]. For example, in a matched cohort study of research participants who use drugs and the general population in Ontario, it was found that PWUD access ED and hospital services seven to eight times more frequently than the non-drug-using population, with most use related to mental health and addiction-related complications [10].

Given that ED are often critical entry points for care, and that PWUD utilize ED at a greater rate than the general population [7–11], understanding ways to improve care for PWUD in these settings remains an important area for intervention. Studies focusing on the experiences of PWUD accessing acute care services have previously highlighted how structural factors within hospital settings (e.g., including abstinence-only drug policies) [12–15], can negatively impact the patient care experience and foster stigmatization of this population resulting in a reluctance to access care in the ED [16, 17] and negative health outcomes [12, 18]. These studies emphasize how these factors not only negatively impact PWUD’s perceptions of care delivery, but also impact the trajectory of patient care, and contribute to future delays in seeking care, discharges, and departures from inpatient care against medical advice [19] and increased vulnerability to drug-related harm [18].

Understanding patient perceptions of and experiences with care delivery in ED represents an important research area within the context of ongoing research focusing on health care providers perspectives [20–25]. Reported challenges in providing adequate care to this patient population in studies with health care providers have included: perceived aggressive patient behaviour or unpleasant patient interactions, concerns about ‘drug-seeking behaviours’, and the burden of balancing limited resources [21]. These challenges also stem from health care providers perceiving themselves to be unprepared to treat patients who use drugs [25, 26], though also frame substance use in stigmatizing terms (e.g., ‘drug-seeking’, ‘aggressiveness’) that locate people who use drugs as the ‘problem’. An evaluation of studies of ED nurses, who are often the first point of contact and provide the most direct care, found that they perceived patients who use drugs as disruptive and affected by a chronic condition that falls outside the ED service mandate [27]. In a study of nurse and physician knowledge and attitudes concerning substance use, gaps were identified in providers confidence regarding the use and dependence of substances other than alcohol. However, respondents identified a shortage of services to treat this patient group as being the primary impediment to adequate management, rather than a lack of training [24]

Negative attitudes and reported challenges in providing care for PWUD are linked to stigma, a well-documented public health issue [28] and barrier to care [16, 21, 29], that is often compounded by discrimination on the basis of race, poverty, and homelessness. Some studies have focused on characterizing the structural factors that foster stigmatization in EDs, moving away from an inter-personal and individual conceptualization of stigma. In a 2013 study conducted in Nova Scotia, Canada researchers described several structures in ED that fostered stigmatization of PWUD who are hepatitis C positive [16]. The findings developed a model of structural stigmatization and indicated that healthcare mandates (e.g., address needs of people who enter the ED while minimizing extended wait times) alongside institutional and departmental constraints and gaps in community-based services forces ED practitioners to make sense of mandates within a setting that is overtaxed and overcrowded [16]. As a result, care providers were influenced to negatively perceive PWUD and their deservingness of care and resulting in stigmatizing experiences and contributing to the reputation of the ED as a stigmatizing space [16]. With attention to these patient and provider dynamics, and the structures that impact care delivery, our research aims to characterize the experiences of PWUD who access EDs to described their experiences and further develop an understanding how PWUD experience stigmatization in ED settings with attention to how these settings reflect and produce structural vulnerabilities contributing to negative health outcomes.

Vancouver, Canada has a large population of PWUD, a significant number of which will access the ED each year. As estimated by a Vancouver-based cohort study, 60% of cohort participants reported accessing the ED in a two year period [8]. Similar to other jurisdictions in Canada [30] and in the United States [31], Vancouver also continues to experience an ongoing overdose crisis [30], resulting in increasing demands for the ED [7].

This paper aims to characterize the contextual social-structural dynamics within the ED as experienced by PWUD to better understand factors associated with leaving against medical advice. By focusing on the experiences of those reporting a poor care outcome, this paper aims to characterize the contextual features within ED that can adversely impact the trajectory of patient care.

## Theoretical framework

This research draws conceptual frameworks of structural vulnerability and intersectionality to understand the dynamics of patient care amongst PWUD in ED settings. Structural vulnerability aims to move away from individualized understandings of health outcomes—and often the blame assigned to individuals for these outcomes—to focus attention on structural determinants of health [32]. This builds on the concept of structural violence, which focuses attention on structural arrangements (e.g., economic opportunity, criminal-legal policies, etc.) that perpetuate adverse outcomes, by further accounting for how these intersect with systemic discrimination (e.g., racism, gender discrimination, ableism) to shape health [32]. The term structural vulnerability refers to one’s location within social structures based on their positionality (as shaped by race, gender, ability, etc.) and how that makes them prone to suffering from the effects of structural violence [32]. A structural vulnerability lens encourages not just a political economic analysis, but also a broader theoretical consideration of how individuals embody the cultural, psychodynamic, symbolic and discursive dimensions of power (symbolic violence) and legitimize neoliberal discourses of individual unworthiness of health and care [33, 34]. In ED settings, the institutionalization of structural violence and internalization of violence can render it invisible through policy (e.g. opioid substitution prescribing policies in EDs) and plays out in everyday interactions, such as between care providers and patients in the ED, in both overt and covert ways [32, 35].

Intersectional approaches further underscore the importance of multi-dimensional and relational processes and interactions occurring between individuals, systems, places and objects across socio-historical contexts creating heterogenous health and drug-related risks and outcomes [36]. An intersectional lens focusses on the inter-related and co-constructed nature of social location and experiences [37] and seeks to tease out the complexity of how social categories such as race, gender, and ability are related to help elucidate the multiple intersecting factors that shape experience [36]. Importantly from an intersectional perspective, social positioning or categories are mutually constructed and interdependent [38], resulting in compounded experiences of, for example, social exclusion, stigma, and drug-related risks [39]. Lastly this approach posits those social categories are not fixed, but rather are “…in a state of becoming, entangled and shaped by social-structural dynamics” [36], such as in interactions between patients and care providers in the ED.

## Methods

This study is situated within a larger program of research that uses ethno-epidemiological methods to examine the experiences of PWUD who left or were discharged from hospital against medical advice [12, 19, 40]. Ethno-epidemiology seeks to harness qualitative methods in relation to epidemiological research infrastructure to examine how social-structural factors influence patterns of drug and health related harms [12]. Qualitative interviews were undertaken in connection with two ongoing prospective cohort studies, the Vancouver Drug User Study (V-DUS) and the AIDS Care Cohort to Evaluate Exposure to Survival Services (ACCESS). These cohorts have been described in detail elsewhere [41] and enroll people who have injected or smoked illicit drugs (other than cannabis) in the past six months, and include both HIV-negative (V-DUS) and HIV-positive (ACCESS) participants. Participants were recruited from the cohort studies by study staff based on whether they had indicated on cohort questionnaires (conducted within the previous two years) that they had left hospital before completing treatment in the previous six-month period. Participants who answered “yes” to the question: “In the past six months, did you leave hospital before your treatment was complete?” were flagged by cohort staff as eligible to participate in this study. Participants were then recruited both over the phone or in-person when they came for their 6-month follow up cohort visits. Indigenous PWUD were oversampled relative to their representation within the drug-using population to better understand previous research finding that they are more likely to leave hospital before completing treatment and experience discrimination in health care settings.

Qualitative interviews were conducted at the cohort research office by the senior author (RM) who has extensive experience in conducting interviews with PWUD. Interviews were conducted from December 2011 to February 2013, and written informed consent was obtained from all participants. There were no refusals to participate and no dropouts. Participants received $20 CAD honoraria following their interviews. An interview topic guide was developed that aimed to facilitate discussion on how contextual factors shaped experiences in hospitals (including ED), leading to discharge against medical advice. Interviews were recorded and later transcribed by a third-party transcription service. RM began data analysis at the project mid-point, and participants were recruited until no new themes emerged from the data, and until a diverse sample of participants with varying demographic characteristics were recruited. Data related to participant’s experiences in the ED were excised by RM from interviews that focused more broadly on hospital care [12], though the overall approach to this sub-analysis was informed by his deep familiarity with the data. This excised data was then reviewed multiple times by VL, RM and SM and a thematic framework was developed based on these readings and with attention paid to the findings from previous analyses [12, 40]. An inductive and deductive approach was then used for the data analysis, which involved coding data to the thematic framework and developing new themes based on further readings of the data [42]. The final themes were then interpreted by drawing on theoretical concepts of structural vulnerability, violence and intersectionality to locate ED experiences within their social-structural contexts. All study activities were approved by the Providence Healthcare/University of British Columbia Research Ethics Board.

## Results

An overview of participant’s demographic information is presented in Table 1. All participants had a history of injection drug use, and twenty-two currently injected drugs. All participants had extensive experience in accessing health services as nearly all reported multiple hospital admissions in the past five years (n = 28). Participants reported a variety of reasons for accessing the hospital including; pneumonia (n = 5), injection related infection (n = 5), physical injury (n = 3) (e.g. being hit by a car, pushed down stairs), osteomyelitis (n = 2), cellulitis (n = 2), endocarditis, blood pressure concerns, hernia, chronic obstructive pulmonary disease, meningitis, bladder infection, seizure, cancer, dehydration, diabetes complications, and kidney infection.

**Table 1:** Participant demographics.

Participant Characteristics	N = 30 (%)
**Age**	
Mean	45.4 years
Range	28–57 years
**Gender**	
Men	16 (53.3%)
Women	13 (43.3%)
Transgender, two-spirit, or non-binary	1 (3.3%)
**Ethnicity**	
White	12 (40.0%)
Indigenous	17 (56.7%)
Other	1 (3.3%)
**Health Status** ^a^^,^^b^	
Human immunodeficiency virus	15 (50.0%)
Hepatitis C Virus	22 (73.3%)
Injection-related infection	12 (40.0%)
Respiratory disease	11 (36.6%)
Cancer	1 (3.3%)
Diabetes	1 (3.3%)
Depression	1 (3.3%)
**Housing**	
Single room occupancy hotels	17 (57%)
Non-market housing	5 (17%)
Emergency Shelters	3 (10%)
Unhoused	5 (17%)
**Drug use (last 30 days)** ^b^	
Crack Cocaine	22 (73%)
Heroin	18 (41%)
Powdered Cocaine	12 (40%)
Prescription opioids	7 (23%)
**Number of hospital admissions in past 5 years**	
One	2 (6.7%)
Two or three	13 (43.3%)
Four or more	15 (50.0%)

^a^ Reported active health conditions at time of interview

^b^ Multiple responses are possible.

The major thematic areas presented here provide details on participants’ perceptions of their experiences in the ED. Reflecting the interviews, these themes overlap to some degree because of cross-cutting experiences of stigmatization that shaped participants’ perceptions of neglect and lack of agency, inadequate pain and withdrawal management and contributed to both their experiences leaving before care was complete and/or their willingness to engage in future care.

### Experiences of stigmatization

Participants reported feeling that they were treated differently in EDs on the basis of their social positions (e.g., drug use, race, socio-economic status, neighbourhood of residence). Most participants emphasized experiences of stigmatization on the basis of their drug use, but articulated other aspects of their social identity that further compounded this stigmatization. As one 39-year-old Indigenous woman seeking care for an injection-related infection explained:

Because under the circumstances having an infection, being a drug user, being, you know, brought in off the street, I guess they tended to like just. My needs aren’t as good as theirs.

In this quote, the participant emphasizes the ED staff’s perception of her identity, as someone who uses drugs, led to her being stigmatized. Importantly, she also articulated how other social identifiers, including her status as someone homeless and with a drug-related health concern (endocarditis), compounded her stigmatization and experience of having her needs dismissed in comparison to the needs of others. Other participants similarly articulated how where they lived and their drug use led them to be stigmatized by health care providers, as emphasized by this participant describing their perspective on being admitted to the ED:

It feels like you’re not important. Like whatever’s wrong with you is all made, it’s all in your head because you’re coming from DTES [Downtown Eastside] because you’re a drug addict. […]There’s already this perception of what you, of what you are and no good for nothing junkie.

[29-year-old Indigenous woman]

In this quote, the participant highlighted how their social identity, as someone who uses drugs, in addition to where they lived, the DTES (an impoverished local drug scene area in Vancouver), resulted in stigmatizing experiences. The quote also reveals how PWUD are perceived as untrustworthy and therefore their experiences and knowledge of their own bodies are not considered useful in informing clinical decisions. Another participant, also discussed their structural vulnerability invoking a broader critique of neo-liberal and individualized notions of self-care. As noted by this participant:

To me I think it was because I’m an alcoholic and an addict. Like cause they probably know because I had to tell them. […]and “Oh she doesn’t care about her body, she doesn’t care about anything, but she’s an addict so we’ll just leave her”. That’s the way I, that’s the way I felt when I was in [hospital name].

[48-year-old Indigenous woman]

This participant links her stigmatization to moralistic views of addiction in society that posits that PWUD do not care for their bodies and are therefore are unworthy of care. Some Indigenous participants also reported feeling discriminated against on the basis of their Indigeneity. As described by one participant prior to receiving a procedure in the ED:

And I’m like well can you explain this to me? On no, no, no just sign it, like I’m some stupid Indian I guess is what the word is.

[41-year-old Indigenous woman]

This participant felt that they were not provided adequate information for a procedure due to the stigmatization they experienced as an Indigenous person. While experiences of racism were not reported by all Indigenous participants, 57% of the sample did identify as Indigenous, suggesting that intersecting racism could compound experiences of drug use related stigma in the ED.

### Experiences of neglect

Participants reported that their perception of stigma were connected to their experiences of neglect in ED settings, including unanswered or dismissed questions related to health care concerns, and a lack of empathy from care providers (e.g. nurses, admitting staff and physicians). This experience of neglect was described by one participant during his initial intake to the ED:

The ambulance took me to [hospital] and the doctor come in, took one look at me like from about thirty feet away and said, there’s nothing wrong with him. He’s just here seeking drugs. Get him out of here. […] What are you talking about I says. I’m not here seeking drugs. I have my own medication right here and I pulled it out of my pocket and held it out and showed him.

[52-year-old white man]

Drug use related stigma led to the participant being considered “drug seeking” and therefore underserving of care. Despite the participants’ insistence and demonstration of care deservingness (showing the doctor their medication), their care was neglected. This sense of neglect was also experienced during repeated ED visits, leading to missed or improper diagnosis of underlying conditions, thus leading to further health complications for some participants. One 29-year-old Indigenous woman described how it took repeated interactions with care providers for her to be diagnosed with meningitis while pregnant:

I found out I was pregnant at the time […] and the doctor looked at me and he told me that there was nothing wrong with me. They said I just had a fever and just to go home. And I was in excruciating pain and I told them that if they didn’t give me any kind of pain control I would cut this baby out of me. So they gave me Dilaudids or whatever […] And the pain was, and of course I didn’t take them by mouth, I fixed them. And then another week went by. I went back to the doctor again.[…] They told me that there was nothing wrong with me. That basically I was just another junkie addict looking for free drugs. They had security escort me out. So, I just didn’t bother to go back.

This participant described how repeated stigmatization of her drug use, compounded with her pain, resulted in neglect and ongoing delays in appropriate treatment. Additionally, the participant’s experience as a pregnant, Indigenous woman bears consideration in terms of how these identities impacted her experience. Previous research has drawn attention to the ways that women who do not conform to familial ideology, including those who use drugs and are pregnant, experience more regulation and stigmatization through medical policies [43]. Moreover, Indigenous women who are pregnant and use drugs can experience multiple complex and intersectional influences, including inter-generational trauma and barriers to care [44].

### Pain and withdrawal management

Stigmatization featured prominently in participants’ narratives of pain and withdrawal management in the ED. Many participants experienced significant acute pain upon presentation to the ED. However, they frequently expressed that their pain was not given serious consideration and that they did not receive adequate treatment or medication. One participant expressed such an experience:

They hooked me up, they gave me an IV, I think. Liquid IV getting water into me or something. But it was useless. The way they treated me. It’s just garbage, man. Like, you’re sitting there in pain and they don’t give you nothing.

[45-year-old white man]

Participants reported that inadequate pain management stemmed from persistent stigmatization on the basis of drug use. Specifically, participants believed that they were predominantly viewed as drug-seeking or ‘dopesick’, and that health care providers withheld pain medication. One participant described this happening when experiencing acute pain in the ED associated with diabetes complications:

[…] I need something. But because I live [in the] DTES, they don’t give me nothing except for, you know acetaminophen.

[41-year-old Indigenous woman]

This participant highlighted how stigmatization of where she lived, led her to experience inadequate care for her pain needs. Some participants acknowledged that policies, or broader structural factors, in the ED (e.g. regulatory policies for pain medication), constrained individual care providers. However, participants’ still perceived that their pain was treated differently in comparison to other patients.

Some participants were hesitant to disclose their drug use in this setting because they were fearful of being stigmatized as drug-seeking, which would impact their quality of care. Participants differentiated between dopesickness (withdrawal), and being ‘really sick’, and expressed that health care providers provided better care when they perceived them to be the latter. As one participant explained:

It actually wasn’t too bad. It wasn’t too bad. They didn’t really treat me too bad because I was, I was literally sick. You know I wasn’t up there just trying to get free dope or something like some people do.

[45-year-old, white man]

In creating a distinction between their situation of being “literally sick” in contrast to a negative framing of people accessing the ED for “free dope”, the participant describes how social violence plays out in everyday interactions in the ED and perpetuates the stigmatization of PWUD.

Some participants had positive experiences and were able to quickly access methadone or morphine in the ED. These participants felt they were able to receive these medications because ED staff could identify they were going through the onset of withdrawal (e.g. chills, sweating), or they felt staff were familiar with their medical history and therefore knew they needed pain medication.

However, other participants expressed barriers and identified structural barriers to receiving adequate withdrawal management. As this participant described:

Respondent: Yeah. The [doctor name] was the one that gives it out and he wouldn’t be there till the next day so I had to wait all night sick until he came around at eight in the morning.

Interviewer: Why do you feel that was the case?

Respondent: Cause [doctor name] is the only one that can give out methadone. There’s no methadone doctors available at that time.

[31-year-old, Indigenous woman]

As this participant describes, there were structural barriers to accessing a methadone prescriber in the ED, leading to unmanaged withdrawal in this setting.

### Leaving against medical advice and (un)willingness to engage in future care

Within the context of participant’s experiences of ongoing stigmatization, neglect, and inadequate pain and withdrawal management, participants reported frequent misunderstandings between health care providers and patients that resulted in some participants leaving against medical advice and before their care was complete. Some participants were asked to leave EDs because they were considered too disruptive and non-compliant:

I was just maybe couple words of profanity now and then I was in so much pain, but I wasn’t being disruptive or disrespectful I thought. […] And so it wasn’t that I was trying to piss her [nurse] off or anything, but I got the feeling from her was that she maybe, I was on drugs or you know I was coming down or I guess some kind of mental problems right […] you know eventually she did kick me out so.

[45-year-old Indigenous man]

This participant described how their experience of distress due to pain was wrongfully attributed to the providers’ perspective that they were experiencing withdrawal or had a mental health challenge, which led to their eventual discharge. While some participants did not directly link their experiences in the ED to leaving the hospital before care was complete, others did report a reluctance to engage in future health services due to their treatment in EDs. When asked about the impact of their experience in the ED on future care seeking, one participant noted:

Demoralizes me. Discourages me from coming to the hospital when I am sick. And that. I would rather let my friends diagnose me and operate on me than going to the doctor [said jokingly].

[29-year-old Indigenous woman].

This participant highlights how they would rather receive care from their friends, people they trust, instead of risking experiences of stigmatization and neglect in subsequent interactions with health care providers.

## Discussion

In summary, this study characterized the experiences of PWUD in ED settings who reported leaving the hospital against medical advice. These findings highlight participants experiences at a specific point in people’s care trajectories that end in being discharged or leaving against medical advice, and represent a point at which it might be possible to intervene to improve care experiences. Interconnected themes included: experiences of stigma, experiences of perceived neglect, inadequate pain and withdrawal management, and unwillingness to engage in future care. While previous studies have similarly identified that PWUD continue to experience stigma in health care settings [13, 14, 18, 45], and unmanaged pain and withdrawal symptoms [18], this study specifically investigates PWUD experiences in EDs who left or were discharged against medical advice, highlighting how structural vulnerabilities are reflected in and produced by policies and interactions in this setting.

Most broadly, these findings point to the need for further consideration of other structural features in ED that shape the experiences of care for PWUD. Other studies have found that PWUD who access the ED are more likely to experience socio-structural barriers, including housing precarity and unemployment, and are not necessarily presenting to the ED for reasons directly related to substance use [11]. Rather, PWUD are presenting to the ED for complex health and social needs, which EDs may be poorly suited to address [11, 46]. Further research has also highlighted the resource limitations providers face in attending to the clinical and social needs of PWUD [21]. For example, care providers have reported limitations in terms of addictions and/or mental health training and specialized staffing supports in the ED (e.g. social workers), as well as a lack of supports in community to which to refer PWUD (e.g. drug treatment spaces in community) [16, 46]. Furthermore, departmental, institutional, and communication structures have also been found to play a role in shaping the stigmatization of PWUD in EDs [16]. For example, a lack of privacy in ED for confidential conversations can affect what information PWUD might provide given the stigmatizing nature of drug use [16, 46].

Overall, these structural factors can create challenges in providing care for PWUD but also represent factors that can be modified through equity-oriented, cultural safety practices and structural competency-informed care to address shortcomings [16]. Equity-oriented care, that aims to mitigate the effect of structural inequities and violence, could also provide valuable ways to improve care for PWUD in ED. A study of an organization-level intervention to promote equity in primary health care settings found that for patients, this intervention predicted greater comfort with and confidence in care [47]. This in in turn predicted greater confidence in managing their own health, and leading to better health outcomes, including fewer depressive and trauma symptoms, less disabling chronic pain and better quality of life [47]. This organizational intervention could also be applied to ED settings [17]. A cultural safety approach to care for PWUD, aims to foster practitioner reflection on the impact of power imbalance and inequitable social relations in health care requiring organizational and cultural shifts in care [48, 49]. Similarly, a structural competency facilitates the leveraging of existing ED infrastructure through a consideration of the structural forces (e.g., hospital policies for prescribing opioid agonist treatments) that shape patient care experiences and behaviour [50].

Specific to this study, structures of pain and opioid maintenance prescribing policies and procedures created barriers to care and fostered perceptions of neglect and stigma. This research aligns with previous studies examining participant’s inadequate pain management for PWUD in hospital settings [18, 51, 52], and increased self-reported pain among ED patients with a history of substance use compared to other patients [53]. Findings from this study build upon previous work highlighting how inadequate pain management is experienced during a variety of clinical encounters [54], including the ED. Other studies have similarly described how inadequately managed pain reinforces marginalization (e.g. stigma from health care professionals, and denial of pain medication) that can lead to risky self-medication [54], which in the context of an ongoing overdose crisis can be life-threatening. This study helps to further characterize a distinct population for which clinical guidelines for pain management in ED settings are needed. These findings point to the importance of addiction medicine training and adequate resources in EDs, including updated clinical care guidelines for PWUD presenting with acute pain to ED [55, 56], and reviewing screening and quality of care measures in order to better address adequate pain management and withdrawal for PWUD in this setting. Intertwined with ineffective pain management, this study’s findings regarding inadequate withdrawal management, further emphasizes the importance of a consideration of withdrawal identification within ED settings and timely access to medications for opioid user disorder (e.g. methadone, buprenorphine). Interventions, such as rapid access addiction clinics or teams embedded in ED settings could ensure patients have timely access to these medications and proper withdrawal care [57].

This study has several limitations, the sample included participants recruited from a cohort accessing hospitals within a particular geographic area (Vancouver, BC), which is shaped by particular contextual forces that influence the drug consumption environments of participants (e.g., availability of supervised consumption services). While these findings may generate insights relevant to other ED settings, they cannot fully account for PWUD experiences in other contexts more broadly. Further, due to recruitment criteria (left or discharged against medical advice) participants experiences are likely negatively biased, and largely do not provide insight into contextual features that encouraged patient retention in hospital. Further, our sample also did not include providers such as, physicians, nurses and administrators, and thus findings may not provide a complete account of the specific contexts under which PWUD left or were discharged against medical advice. Lastly, this data was collected from 2011 to 2013, while more current studies have reported similar results, findings from this study are contextualized within the time period they were collected which may have limitations in implementation.

In conclusion, these findings highlight how structural considerations in ED settings, such as how pain and withdrawal are managed, can negatively impact the quality of care for PWUD, lending to care dissatisfaction, ongoing stigmatization and contributing to instances of discharge and leaving hospital against medical advice. Findings highlight important considerations regarding approaches to pain management for PWUD, improved access to opioid agonist treatment in the ED, and the overall need for a structural competency informed approach to care delivery for PWUD in this care setting.

## Supporting information

S1 File Inclusivity in global research. (DOCX)
